# Edge Effects Are Important in Supporting Beetle Biodiversity in a Gravel-Bed River Floodplain

**DOI:** 10.1371/journal.pone.0114415

**Published:** 2014-12-29

**Authors:** Simone D. Langhans, Klement Tockner

**Affiliations:** 1 Eawag, Swiss Federal Institute of Aquatic Science and Technology, Überlandstrasse 133, 8600 Dübendorf, Switzerland; 2 Institute of Integrative Biology, ETH Zürich, 8092 Zürich, Switzerland; 3 Leibniz-Institute of Freshwater Ecology and Inland Fisheries, Müggelseedamm 310, 12587 Berlin, Germany; 4 Institute of Biology, Freie Universität Berlin, Berlin, Germany; Università degli Studi di Napoli Federico II, Italy

## Abstract

Understanding complex, dynamic, and diverse ecosystems is essential for developing sound management and conservation strategies. Gravel-bed river floodplains are composed of an interlinked mosaic of aquatic and terrestrial habitats hosting a diverse, specialized, and endangered fauna. Therefore, they serve as excellent models to investigate the biodiversity of multiple ecotones and related edge effects. In this study, we investigated the abundance, composition, richness, and conservation status of beetle assemblages at varying sediment depth (0, 0.1, 0.6 and 1.1 m), distance from the channel (1, 5, 20, and 60–100 m, and 5 m within the riparian forest), and time of the year (February–November) across a 200 m-wide gravel bar at the near-natural Tagliamento River (Italy), to detect edge effects in four floodplain ecotones: aquatic-terrestrial, forest-active floodplain, sediment-air, and sediment-groundwater. We used conventional pitfall traps and novel tube traps to sample beetles comparably on the sediment surface and within the unsaturated sediments. We found a total of 308 beetle species (including 87 of conservation concern) that showed multiple, significant positive edge effects across the floodplain ecotones, mainly driven by spatial heterogeneity: Total and red list beetle abundance and richness peaked on the sediment surface, at channel margins, and at the edge of the riparian forest. All ecotones possessed edge/habitat specialists. Most red list species occurred on the sediment surface, including five species previously considered extinct – yet two of these species occurred in higher densities in the unsaturated sediments. Conservation and management efforts along gravel-bed rivers must therefore promote a dynamic flow and sediment regime to create and maintain habitat heterogeneity and ecotone diversity, which support a unique and high biodiversity.

## Introduction

Braided gravel-bed rivers are widespread in temperate piedmont and mountain-valleys [1]. In their pristine state, they consist of complex mosaics of aquatic and terrestrial habitats created through frequent flow and flood pulses [2]. Receding floods and dry spells expose vast areas of bare gravel that form boundaries – also called ecotones or interfaces – between land and water [3].

**Figure 1:**
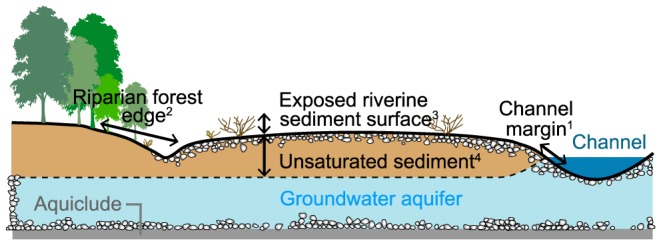
Location of the four investigated floodplain ecotones. Cross-section of a floodplain gravel bar indicating the location of the four consequential ecotones included in the study: ^1^the channel margin (aquatic – terrestrial ecotone), ^2^the edge of the riparian forest (forest – active floodplain ecotone), ^3^the sediment surface (sediment – air ecotone), and ^4^the unsaturated interstitial sediment below the sediment surface and above the groundwater table (sediment – groundwater ecotone).

On a finer-scale, exposed gravel bars embed secondary ecotones (*sensu* [4]) including i) the channel margin (i.e., the aquatic-terrestrial ecotone [3]), ii) the edge of the riparian forest (i.e., the forest-active floodplain ecotone [5]), iii) the sediment surface (i.e., the sediment-air ecotone [6]), and iv) the unsaturated layers below the surface but above the groundwater (i.e., the sediment-groundwater ecotone [7]) (Fig. 1).

Ecotones control the spatio-temporal distribution and dynamics of many species [5], [8]. This can be attributed to so-called edge effects. Edge effects include any environmental attribute that is altered as a result of being at, or in proximity to the border between two habitats. They can be abiotic changes and changes to behavior or ecological processes [9], [10]. Edge effects frequently emerge at interactive boundaries (*sensu* [4]) between contrasting habitat types that may lead to increased population density and species richness. However, the effect of interactive boundaries and the associated edge effects on species density and richness remains a subject of debate [10]–[15].

A possible way to investigate edge effects is considering species that are primarily associated with an ecotone (aka edge specialists). The edge-effect concept has been mostly applied to explain assemblage structures of birds at forest edges [16]. However, it may be an excellent approach to investigate the response of other taxa than birds to advance ecotone concepts, and to support conservation efforts especially in landscape mosaics.

Exposed riverine sediments as well as the fringing riparian forest possess an abundant, diverse, and highly specialized beetle fauna that is adapted to flood disturbances and extreme environmental conditions [17]–[19]. Many of these species are listed as endangered. At the same time, knowledge about beetle assemblages within the sediment (the so-called hypogeic fauna) is scarce, despite extensive layers of unsaturated sediments being a key feature of braided rivers. For example, along the Tagliamento River in Italy the 38.7 km^2^ of exposed sediments are associated with approximately 58 million m^3^ of unsaturated sediments, assuming an average depth of the unsaturated zone to be 1.5 m. Even if only parts of this volume are accessible to the riparian fauna, it is the most extensive habitat along braided rivers, with likely important functions for population dynamics and ecosystem processes [20].

The rich beetle fauna inhabiting exposed and unsaturated sediments [20] provides an excellent opportunity to test edge effects on species diversity and assemblage structures at relevant spatial scales. At the same time, gravel-bed rivers are among the most human-altered ecosystems, with severe consequences for biodiversity [21]. Therefore, knowing whether ecotones boost floodplain diversity is challenging both from a theoretical point of view as well as from a conservation perspective.

We predicted that beetles respond with increased abundance and richness to the availability of floodplain ecotones. This is considered a consequence of the high degree of specialization of most beetles found on exposed river sediments, the close proximity of the exposed sediments to a highly productive and diverse riparian zone [22], and the foraging behaviour of predatory beetle species along channel margins [23]. We expected similar population patterns for species of conservation concern, because exposed gravel habitats are among the most threatened ecosystem type today.

To test these predictions, and to better understand the underlying factors that structure riparian beetle communities, we addressed the following questions:

1. Are the four studied ecotones promoting beetle abundance and richness of exposed river sediments through positive edge effects?
2. If so, what role do edge specialists play?
3. How important are the four ecotones for species of conservation concern?
4. Do spatial, environmental, or temporal variables most strongly define species distribution patterns on exposed river sediments?

## Materials and Methods

### Study site

Our study site was located within the island-braided section of the 7^th^-order gravel-bed river Tagliamento (Italy). The Tagliamento originates in the southern fringe of the European Alps and flows almost unimpeded by dams for 172 km to the Adriatic Sea. Consequently, the upper and middle reaches feature an near-natural sediment regime driven by frequent flow and flood pulses [24]. The island-braided section (river-km 79.8–80.8; 135 m a.s.l.) which extends to 1.5 km width, contains a spatially complex and temporally dynamic habitat mosaic dominated by extensive areas of exposed river sediments [25]. There, the 800 m-wide active tract is fringed by a ribbon of intact riparian forest [26]. Distinct spatial heterogeneity and high disturbance frequency support a rich and specialized beetle fauna [27]–[29].

**Figure 2:**
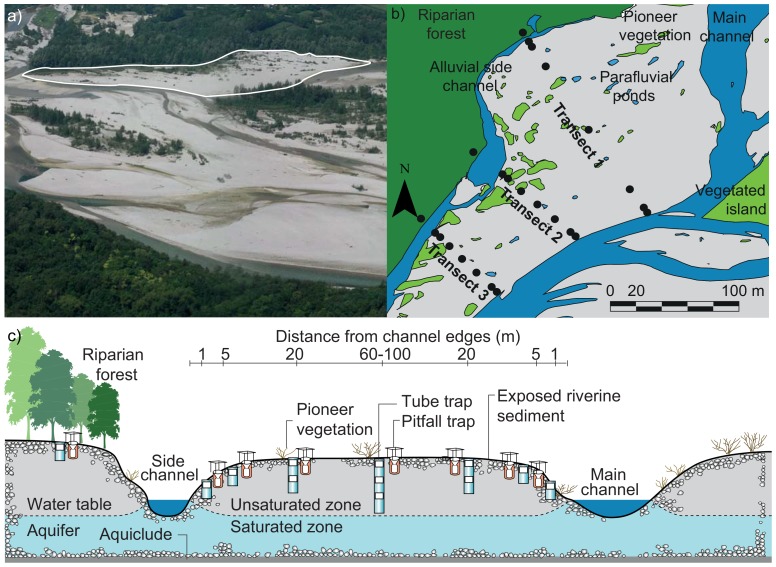
Investigated gravel bar and study design. a) Investigated gravel bar (contour in white; flow direction from right to left), b) survey design including three replicated transects with indications of the eight trap locations at 1, 5, and 20 m from the left channel margin, 1, 5, and 20 m from the right channel margin, and in the center of the gravel bar (60–100 m distance from each channel), and at the edge of the riparian forest (5 m within the vegetation), and c) cross section of a transect (adapted from [20]).

We selected a 200 m-wide, and 500-m long gravel bar fringed by a 20 m-wide channel on the left bank, and by a small alluvial groundwater channel (width: ≤5 m) and the riparian forest on the right bank (Fig. 2a). Both channels featured permanent water flow throughout the study. Sediments on the gravel bar consisted of gravel and pebble [30] with patches of sand along the alluvial groundwater channel. The gravel bar that was studied is usually completely inundated twice a year, when snowmelt in spring and rain events in autumn trigger bank-full floods. 2005 was an exception with no major flood event. Smaller fluctuations in the water level and therewith partial inundation of the exposed gravel occur throughout the year [24].

### Sampling design and beetle trapping

We used two trapping methods that allowed quantitative comparisons of beetle catches: pitfall traps on the sediment surface [31], and tube traps [20] in the unsaturated sediments. Both trap types measure activity density [32], [33] with comparable sampling efficacy [20].

We installed tube traps and pitfall traps in pairs at eight locations along three transects: at 1, 5, and 20 m from the left channel margin, in the middle of the gravel bar (at 60–100 m from both channel margins), at 1, 5, and 20 m from the right channel margin, and 5 m within the riparian forest (Fig. 2b). Defined by the groundwater table, we used short (0.5 m), middle (1.0 m), or long (1.5 m) tube traps with one (at 1 and 5 m distance from the channels), two (at 20 m distance), or three stacked sampling compartments (at 60–100 m distance), respectively (Fig. 2c; [20]). Transects were installed at approximately 60 m distance from each other to ensure independence.

To minimize disturbance effects on the beetle fauna, tube traps were installed four months before the experiment started. At monthly intervals, from February until November 2005, we exposed pitfall traps for approximately one week, and tube traps for approximately two weeks to adjust for lower beetle abundances in the sediments. Sampling durations varied among months to avoid flooding of the traps. Nevertheless, we lost two tube-trap samples during the sampling campaign in February, two during April, and seven during the campaign in May. Samples were sieved through a 100 µm-mesh screen, and beetles were identified to species level by M. Kahlen (Tiroler Landesmuseen, Innsbruck), using published keys and reference collections.

Permission to install tube traps within the floodplain gravel bar was obtained from the municipality of Pinzano al Tagliamento (Italy). No specific permission was required to collect riparian beetles, because they are not protected in the region.

### Habitat variables

**Figure 3:**
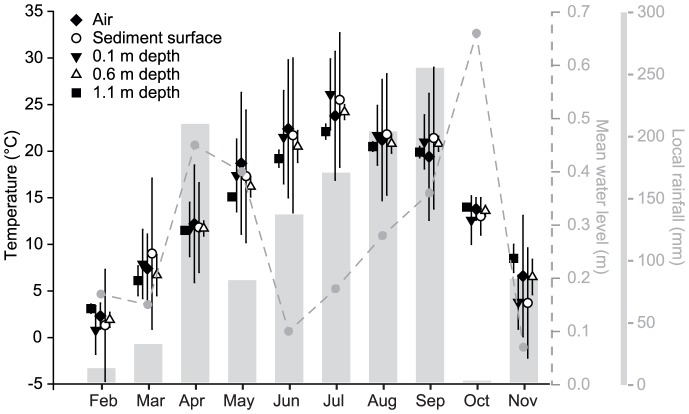
Meteorological characteristics measured during the sampling period across the studied gravel bar (Tagliamento River, Italy; mean ±1 SD where applicable).

We continuously recorded air temperature, rainfall, and temperature at the sediment surface and at three different sediment depths (0.1, 0.6, and 1.1 m) on the sample site with a HOBO micro weather station (for rainfall; onset, Pocasset, MA, USA), a HOBO Pro RH/Temp data logger (for air temperature; onset, Pocasset, MA, USA), and Vemco Minilog data logger (for sediment surface and subsurface temperatures at each sampling location; MINILOG12-TR-40/+50−064K, Vemco, Nova Scotia, Canada), respectively. The surface water level was recorded continuously 500 m downstream of the studied gravel bar at Villuzza (Fig. 3).

### Abundance, richness, and species of conservation concern

To test the role of the four ecotones on assemblage structure, we calculated relative abundance (measured as individuals (ind) m^−2^ day^−1^), total species richness (per trap) and relative abundance of species with conservation concern (ind m^−2^ day^−1^) for each replicated trap location and month. To compare surface and subsurface samples, we standardized abundances according to the duration of exposure and opening area of pitfall- and tube traps. The area of the opening for tube traps was the sum of the two opposite square holes (0.048 m^2^), and for pitfall traps the circular opening area of the funnel (0.018 m^2^) [20]. We defined beetles with conservation status according to the red list of Germany [34], since no such list was available for the region studied.

We used relative abundance, species richness and relative abundance of species with conservation concern as response variables in separate linear mixed models (LMMs). LMMs can accommodate repeated measures, random factors, and fixed factors, and can deal with unbalanced sampling designs and/or missing values [35]. The parameters of the LMMs were the fixed factors ‘depth’ with four levels (0, 0.1, 0.6, and 1.1 m) and ‘distance from channels’ with five levels (1, 5, 20, and 60–100 m, and 5 m within the riparian forest), the random factor ‘transect’, and the repeated-measures factor ‘month’. We first defined the most appropriate covariance structure of the model by choosing the one with the smallest Akaike's information criterion (AIC) (first order autoregressive (AR1) for total beetle abundance and abundance of red list species only, and unstructured for species richness). We then estimated the parameters of our statistical model using restricted maximum likelihood (REML) with 100 iterations and significances of pairwise comparisons with Sidak post-hoc tests. All LMMs were carried out in SPSS (ver. 19.0/SPSS Inc., Illinois, USA) using a type III sums-of-squares F test with significance levels set at *P*≤0.05.

### Assemblage composition

We investigated whether the composition of the total species assemblage and red list species assemblage was a function of spatial position (reflecting the influence of biotic processes), season (reflecting the life-cycles of species), or environmental variables (reflecting the small-scale habitat), using partial redundancy analyses (RDA) with subsequent variance partitioning [36], [37] in R with the package ‘vegan’ [38], [39].

We constructed two species matrices (one for all species, and one for the red list species only), using relative abundances standardized for trap type (ind m^−2^ day^−1^). We transformed the abundance data matrices such that the RDA would yield the Hellinger distance between sites. Hellinger distance has been shown to be more appropriate for ordination of species abundance data containing many zeros, than Euclidean distance [40]. We then constructed a variable matrix retaining the explanatory variables sediment depth, distance to the channel margin, and distance to the riparian forest (spatial variables), the four seasons (temporal variables; winter (November and February data), spring (March, April, May), summer (June, July, August), autumn (September, October)), and sediment temperature, rainfall, and water level (environmental variables). We excluded the variable air temperature from the RDA, because it was highly correlated with sediment temperature (Spearman rank's correlation: ρ>0.7). We kept rare species (i.e., sampled only once) in the data matrices, since they are better indicators of ecosystem stress than are common species [41], [42] and can add significant site-specific information [43].

Finally, we used the species matrix and variable matrix for two different analyses: 1) partial RDA (pRDA) of the two species matrices constrained by the variable matrix, accounting for variation explained by the transects; and 2) variance partitioning among the spatial, temporal, and environmental gradients, for both species data sets based on the pRDA. In step one, we estimated the statistical significance of each explanatory variable with 1000 Monte-Carlo permutations, using forward selection routines [44] with alpha set at 0.001 to account for type one errors [45], due to the many covariates and the size of the data set. For step two, we split the variable matrix into three separate matrices containing spatial, temporal, and environmental variables only. All response variables were log(x+1)-transformed prior to analyses to improve normality. Note that the spatial variables, i.e., edge proximity, did not only represent geographic information [46] but was also a proxy for environmental conditions.

## Results

### Species distribution

**Figure 4:**
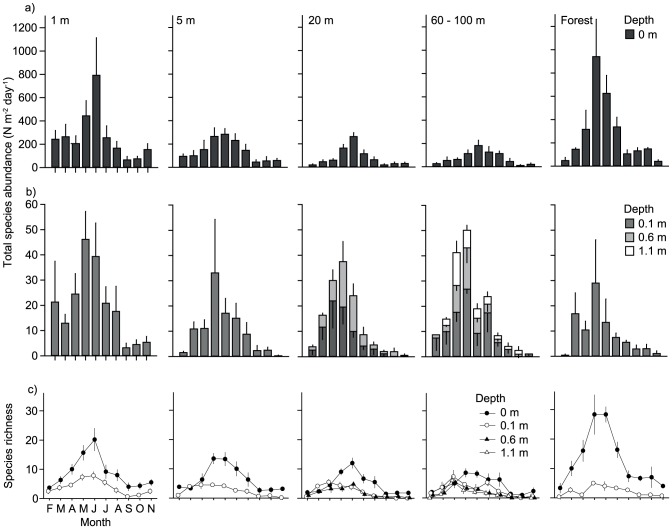
Monthly beetle abundance and beetle species richness patterns. Monthly changes in a, b) beetle abundance (ind m^−2^ day^−1^) and c) beetle species richness at four different depths (0, 0.1, 0.6, and 1.1 m), and at five distances across a large gravel bar (1, 5, 20, and 60–100 m distance from the channel margin, and at the edge of the riparian forest). Data are monthly means +/− SE.

We collected a total of 7666 individual beetles (5479 individuals in 240 samples on the surface and 2187 individuals in 349 samples within the sediment; S1 Table). Density ranged from 0 to 333 individuals per pitfall trap on the sediment surface (mean ± SE: 23.0±2.2 ind trap^−1^; N = 240), and from 0 to 78 individuals per trap within the sediment (mean ± SE; 6.3±0.6 ind trap^−1^; N = 356). Relative beetle abundance, standardized for trap dimensions and exposure time, ranged from 0 to 2355.6 ind m^−2^ d^−1^ on the sediment surface (mean ± SE: 167.7±15.6, N = 240) and from 0 to 106.6 ind m^−2^ d^−1^ within the sediment (mean ± SE: 9.2±0.8, N = 356) (Fig. 4a,b). On the sediment surface, relative abundance was lowest in September (mean ± SE: 55.2±11.9, N = 24) and highest in June (mean ± SE: 435.6±92.8, N = 24), whereas within the sediment abundance was lowest in November (mean ± SE: 1.1±0.5, N = 36) and highest in May (mean ± SE: 22.4±3.9, N = 36) (Fig. 4a,b).

**Figure 5:**
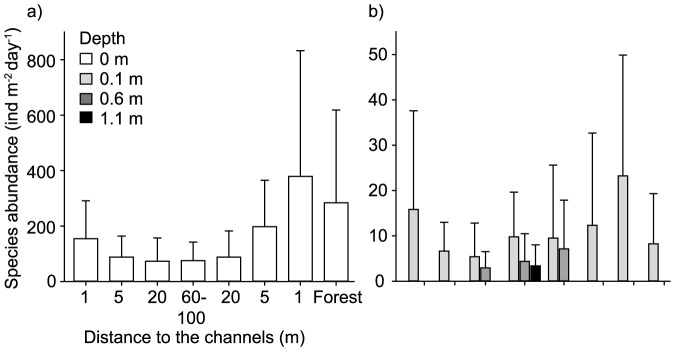
Total species abundance (ind m^−2^ day^−1^; ±1SD) a) on the sediment surface and b) within the unsaturated sediments (pooled over all months; N = 28–30).

Sediment depth and distance to the channels (and the riparian forest) had significant effects on relative beetle abundance (F_3, 116.9_ = 64.4, P<0.001, N = 594 and F_3, 116.6_ = 5.0, P<0.001, N = 594, respectively; Figs. 4a,b, and 5). Abundance was highest at the surface and at 1 and 5 m distance to the channels (including forest samples), and significantly higher than at 0.1 m depth and in 20 and 60–100 m distance. Abundance at 0.6 and 1.1 m depth was significantly lower than at 0.1 m depth.

### Species richness

We identified a total of 308 beetle species from 34 families (S1 Table). Horizontally, species richness peaked at 1 m distance from the channels (170 species; pooled over all months and all replicated traps) and at the forest edge (155 species). Richness decreased towards the center of the gravel bar (136, 104, and 73 species, respectively; Table 1). Vertically, a total of 271 species occurred at the sediment surface, and 117, 45, and 19 species in the subsequent sediment layers (Table 1).

**Table 1:** Species richness at each location and depth category pooled over all months (N = 28–240).

	Distance from channels (m)					
Depth (m)	1	5	20	60–100	Forest	Total
0	137	114	73	44	147	271
0.1	78	52	43	38	29	117
0.6			38	19		45
1.1				19		19
Total	170	136	104	73	155	

Of the 308 species, 181 species were only caught on the sediment surface (at the sediment-air ecotone), 35 species only within the subsurface sediment (at the sediment-groundwater ecotone), 32 species only at channel margins (at the aquatic-terrestrial ecotone), and 72 species only at the edge of the riparian forest (at the forest-active floodplain ecotone; S1 Table). Thereof, we identified those species to be true edge specialists that are not expected to occur in the habitat patch at “the other side” of the edge: Nine edge specialists at the sediment-air ecotone, i.e. those of the 181 species that can not fly, and therefore do not occur in the airscape (S1 Table); 35 edge specialists at the sediment-groundwater ecotone, because none of the 35 species was a stygophile species inhabiting the groundwater beneath the unsaturated sediments; and 28 edge specialists at the aquatic-terrestrial ecotone, excluding the aquatic species *Potamonectes depressus elegans, Orectochilus villosus, Hydraena sp.*, and *Ochthebius nobilis* from the 32 species only found at the channel margin, as they may also occur in the river channel. Note that some of the 72 species at the forest-active floodplain ecotone may not be true edge specialists, but merely habitat specialists, since they may also occur within the upland forest, which we have not sampled. Hence, we refer to these species as habitat/edge specialists.

Approximately 30% of all species were considered rare and were only caught once throughout the sampling period. On the sediment surface and within the sediment, the majority of species were staphylinids and carabids (35% and 29%, and 42% and 24%, respectively).

Sediment depth and distance to the channels (and the riparian forest) showed significant effects on species richness (depth: F_3, 48.5_ = 59.2, P<0.001, N = 594; distance: F_4, 47.3_ = 17.2, P<0.001, N = 594) (Fig. 4c). Species richness was significantly higher on the sediment surface than in the sediment, and at 1 m and at the edge of the riparian forest than at 5 m distance to the channels. Species richness was lowest towards the center of the gravel bar (20 and 60–100 m distance) (Fig. 4c). We found a significant interaction effect between depth X distance (F_5, 1165_ = 1.4, P = 0.001, N = 594). This effect was due to a much sharper decrease in species richness in forest edge samples from the sediment surface towards 0.1 m depth, than in all other locations.

### Distribution of species with conservation concern

A total of 87 species (i.e. 28% of the collected species) are listed as threatened (S2 Table). Forty-three species are restricted in their occurrence to the sediment surface, eight to the subsurface sediments, ten to the edge of the riparian forest, and seven to channel margin (S2 Table). Additionally, *Bembidion foraminosum, Drasterius bimaculatus, Colon fuscicorne, Chelonoidum latum*, and *Brachygluta trigonoprocta* were previously considered regionally extinct (S2 Table).

**Figure 6:**
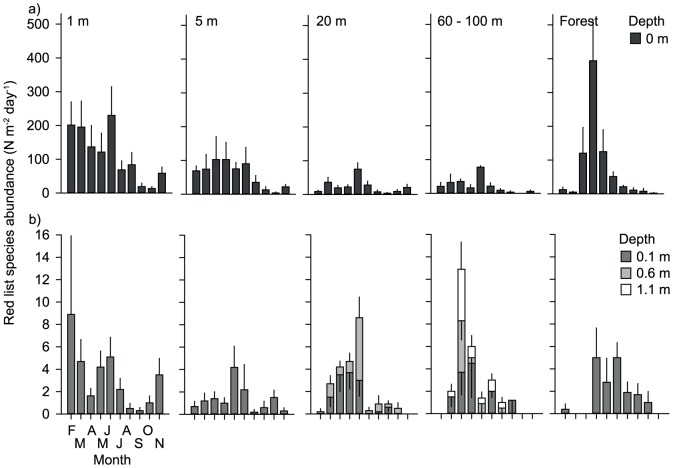
Abundance patterns of beetles with conservation concern. Monthly changes in abundance of beetles with conservation concern (ind m^−2^ d^−1^) a) at the sediment surface, and b) at 0.1, 0.6 and 1.1 m depth and at five locations across the gravel bar (1, 5, 20, and 60–100 m distance from the channel margin and at the edge of the riparian forest). Data are monthly means +/−1SE.

Red list species were significantly affected by sediment depth and distance to channels and the riparian forest (depth: F_3, 131.4_ = 60.6, P<0.001, N = 594; distance: F_4, 131.3_ = 6.0, P<0.001, N = 594) (Fig. 6a,b). They were significantly more abundant at the sediment surface than in the subsurface sediments, and at 1 and 5 m (including forest samples) than at 20 and 60–100 m distance to the channels.

### Assemblage composition

Partial RDAs calculated the effect of the sample locations on the composition of all beetle species as well as the red list species assemblages, when the transect effect was removed. A total of 13.1% and 13.5% of the variation in the total species matrix and the red list species matrix, respectively, were explained by the retained variables (Table A in S3 Table, Table B in S3 Table). The first seven and five canonical axes, respectively, were significantly related to the data. In the total species matrix, 5% of the explained variation was purely spatial, 3% purely temporal, and 2% purely environmental (all Ps≤0.001; S4 Table). In the red list species matrix, 5% was purely spatial, 2% purely environmental, and 1% of the explained variation purely temporal (all Ps≤0.001; S4 Table). In summary, only a small fraction of the variation was explained by the variables that we have quantified.

**Figure 7:**
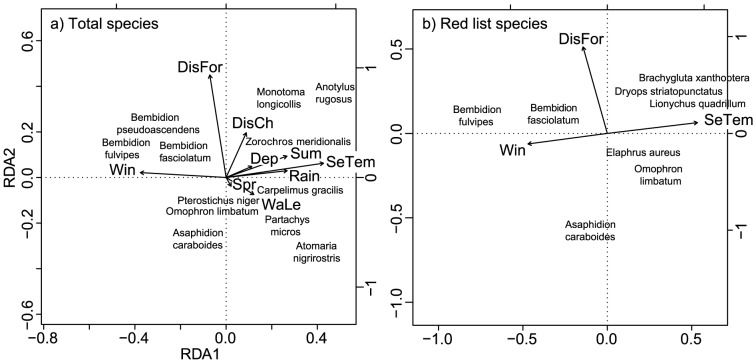
Partial redundancy analysis. Partial redundancy analysis (pRDA) results for a) total species distribution, and b) red list species distribution and spatial, temporal, and environmental variables. Only significant variables (S5 Table) and species that discriminate well are shown. Variable abbreviations: Dep  =  depth, DisCh  =  distance to channels, DisFo  =  distance to the riparian forest, Win  =  winter, Spr  =  spring, Sum  =  summer, WaLe  =  water level, SeTem  =  sediment temperature.

All variables except the temporal variable ‘autumn’ were found to be significant in structuring the distribution of the total species assemblage (Fig. 7a, S5 Table). The RDA discriminated well for twelve species. *Bembidion fulvipes* (Carabidae), *Bembidion pseudoascendens* (Carabidae), and *Bembidion fasciolatum* (Carabidae) were clearly associated with ‘winter’, *Atomaria nigrirostris* (Cryptophagidae), *Paratachys micros* (Carabidae), *Omophron limbatum* (Carabidae), *Pterostichus niger* (Carabidae), and *Carpelimus gracilis* (Staphylinidae) with ‘water level’, and *Zorochros meridionalis* (Elateridae) and *Monotoma longicollis* (Monotomidae) with ‘distance to the channel margins’. *Asaphidion caraboides* (Carabidae) showed a negative association with the two spatial variables: ‘distance to the forest’ and ‘distance to the channels’.

The three variables ‘distance to the riparian forest’, ‘sediment temperature’, and ‘winter’ were significant in structuring the distribution of red list species (Fig. 7b, S5 Table). Of the eight species for which the RDA discriminated well, *Bembidion fulvipes* and *Bembidion fasciolatum* (both Carabidae) were clearly associated with ‘winter’, and *Dryops striatopunctatus* (Dryopidae), *Brachygluta xanthoptera* (Staphylinidae), and *Lionychus quadrillum* (Carabidae) with ‘sediment temperature’ and ‘distance to the riparian forest’.

## Discussion

Edge effects have been discussed broadly albeit contentiously in the scientific literature. If present, they considerably shape species distribution patterns in landscape mosaics [3], [8], and therefore help inform population management and biodiversity conservation. In the present study, we detected multiple positive edge effects across the investigated floodplain ecotones, mainly driven by spatial variation: Total species abundance and species richness peaked on the sediment surface, at channel margins, and at the edge of the riparian forest, and all four ecotones (aquatic-terrestrial, forest-active floodplain, sediment-air, and sediment-groundwater) contained a considerable proportion of edge/habitat specialists. Similarly, species of conservation concern peaked on the sediment surface, including five species previously considered extinct. Although the unsaturated sediments did not exhibit a positive edge effect, they inhabited a considerable number of beetles including higher numbers for two of the extinct species than on the sediment surface.

### Beetle assemblage patterns across floodplain ecotones

High abundance and diversity at ecotones can emerge due to a range of factors. In our study, channel margins likely sustained predatory species, mostly carabid and staphylinid beetles that forage on emerging aquatic and stranded aquatic and terrestrial insects [23]. Life cycles of these species are often synchronized with emergence patterns of aquatic insects [47], resulting in high beetle abundances during May and June, as confirmed in this study.

High beetle abundance and diversity at the edge of the riparian forest were likely a consequence of the availability of a diverse array of habitats and ecological services in this ecotone [48]. Environmental heterogeneity may have also promoted many specialist species (72) at the forest-active floodplain edge. Similarly, Steward *et al.* [29] found a diverse and distinct arthropod assemblage within the riparian zone. This edge effect seems not to be exclusive for riparian forests: increased carabid diversity along forest-grassland ecotones, attributed to the occurrence of edge specialists, has been found in a series of previous studies [13], [14], [49].

The gradual decrease in species abundance and richness with increasing sediment depth (and distance to the channel margin) is attributable to species-specific traits. About 60% of the species seemed to be restricted in their occurrence to the sediment surface. A considerable number of edge specialists (28; about 10% of all species) exhibited the same affinity to channel margins. The observed horizontal and vertical decrease in abundance and richness therefore emphasizes the effect of species-specific behavior on assemblage structure, and the importance of small-scale gradients in shaping populations. Such gradients should be taken into account when sampling a given habitat, and when modeling animal movement, species distribution, or diversity pattern in heterogeneous landscapes. These microscale effects of distance are likely to affect large-scale species and population patterns [50].

Decreasing species abundance and richness observed with increasing sediment depth may indicate that the unsaturated sediment should not be considered an ecotone but rather a habitat patch (*sensu* [51]) between the sediment surface and the groundwater. Alternatively, the unsaturated sediments could be considered a noninteractive ecotone (*sensu* [4]) – a hypothesis supported by the considerable proportion of edge specialists (11% of the species) found in this habitat. Further studies are required to accept or reject this hypothesis.

### Response of beetles to spatial, temporal, and environmental heterogeneity gradients

Spatial, temporal, and environmental variables, single and in concert, explained a significant albeit rather small amount of the variation in the distribution of all species and species of conservation concern, respectively. Spatial variables alone accounted for a third of the variation in total abundance and in abundance of species of conservation concern (each 5% of explained variation), while temporal and environmental variation accounted for even less. Hence, spatial structuring that develops due to biotic processes such as predation, competition, dispersal, disturbance, or disease appears to have the greatest effect on the composition of floodplain beetles in our study area. Moreover, since the spatial variables represented edge proximity (and not merely geographic information as in the majority of pRDA studies [46]), this finding may be an additional indicator for the structuring effect of ecotones for beetle communities across the river-floodplain mosaic.

The significant correlation between abundances and the three variable types, particularly with ‘distance to the riparian forest’ (spatial), ‘temperature’ (environmental), and ‘winter’ (temporal) (Fig. 7a,b), suggests complex distribution patterns in these beetle assemblages. This was especially prominent for carabid and staphylinid beetles. Some species of these families were correlated with ‘winter’ (Fig. 7a,b), indicating that they are spring breeders [52], [53] that overwinter as adults within the unsaturated sediment layers. Indeed, species of *Bembidion*, and several staphylinid beetles do so usually higher up at drier locations [54], [55].

Other species were associated with specific locations across the gravel bar or changes in water level. This may indicate feeding preferences, e.g. along channel margins being predators [23], [47], or in or close to vegetated areas feeding on organic matter. *Asaphidion caraboides*, for example, showed a clear negative association with the spatial variable ‘distance to the riparian forest and channels’. This may indicate that *A. caraboides* likely occurs close to those ecotones. Indeed, this species seems to prefer riparian areas close to shorelines that consist of fine sand [56]. Another example is the association of the red list species *Dryops striatopunctatus* with ‘sediment depth’ (not significant and therefore not shown in Fig. 7b), which hints at the species' preference for aquatic or semi-aquatic habitats. Such habitats can be found in the deeper sediment layers of the investigated gravel bar.

Distribution patterns of other beetles are difficult to explain. This is due to the fact that information on species-specific habitat preferences or life-history strategies is still scarce or missing. This is especially true for many beetles living on exposed river sediments and in adjacent ecotones [27], [57]. Therefore, our results help describe species-specific behaviour which can directly translate into targeted management and conservation.

A large fraction of the variation in both total and red list species composition remained unexplained by the spatial, temporal, and environmental variables. This is not uncommon in ecological studies [36], [58]. The reason for this could be the poor fit of ecological data to the models underlying common ordination methods, resulting in the creation of meaningless distortion axes that can contribute substantially to the total variance [59]. Moreover, the large residual variation may have been due to stochastic variation in assemblage composition, overlooked factors not included in the study, and/or sampling error [60]. Spatially distinct information on interstitial spaces, soil moisture, or sediment organic matter content may have helped to explain more of the variation in species assemblages within the sediment. However, acquiring this information would have disturbed the habitats and significantly modified beetle behavior. Finally, some beetle species present at the study site may not have been detected with the sampling methods used. Pitfall traps and most likely tube traps, over-represent large and mobile species and underrepresent smaller, cryptic beetles [61].

### Implications for conservation

Extensive taxonomic surveys along the Tagliamento River in Italy have previously recorded approximately 500 beetle species associated with exposed sediments [27], [28]. Gravel-bed rivers are heavily impacted throughout Europe [21], therefore a high proportion of these species must be considered threatened [27].

In the present study, we found 87 species with conservation concern (28% of all species) across the four studied ecotones. Very similar numbers have been found in the UK, where near-natural exposed sediment areas were associated with 81 beetles considered nationally scarce, of which 42 were floodplain specialists [18]. Additionally, we found five species that were considered regionally extinct (according to the red list of Germany). Therefore, our findings affirm previous studies [18], [62] that attribute a high conservation value to near-natural exposed river sediments.

Novel to the literature of exposed river sediments are edge effects on species of conservation concern. They occur at high abundance and richness at the sediment surface, at channel margins, and at the edge of the riparian forest, with a considerable proportion of edge/habitat specialists. Hence, all four studied ecotones, including the unsaturated sediments, seem to be significant for conservation, serving as a source habitat (i.e., refugium) for dispersal and recolonization processes following disturbance events [13], [14].

Although 75% of the red list species were floodplain specialists, many of them were associated with distinct spatial, temporal, and environmental variables. It indicates that these species differ considerably in life-history and small-scale habitat preference.

Habitat diversity is very pronounced along near-natural gravel-bed rivers. Therefore, management and conservation efforts should 1) target floodplain ecotones, including the unsaturated sediments as an entity, since they create and maintain high floodplain biodiversity, and 2) promote natural processes, including a natural flood and sediment regime that create habitat heterogeneity at different spatial scales [19], [63]. Thereby, a catchment perspective needs to be adopted because sustainable meta-populations of floodplain specialists rely on unconstrained floodplain segments, multiple gravel bars [18], and associated ecotones.

## Supporting Information

S1 Table **Full taxa list and pooled samples of beetles caught on the sediment surface and within subsurface sediments across a 200 m-wide gravel bar at the Tagliamento River (Italy).** * species were only sampled on the sediment surface and can not or only partly fly (M. Kahlen, personal communication).
(PDF)

S2 Table **Species (No. of individuals) captured on the sediment surface (Surf.), within the subsurface sediments (Sed.), only at channel margins and at the riparian forest edge, respectively, that are of conservation concern (Status) according to Binot et al.** [1] **.** Re  =  regionally extinct, CR  =  critically endangered, EN  =  endangered, VU  =  vulnerable, NT  =  near threatened, R  =  rare.
(PDF)

S3 Table **Eigenvalues of the partial RDA, and the cumulative percentage of variance explained by significant axes A) for all species and B) for species of conservation concern.**
(PDF)

S4 Table **The contribution of spatial, environmental, and temporal variables to the variation in the two species matrices (all species and species of conservation concern only) explained by the partial RDA.** R^2^ adjusted with Ezekiel's formula [1].
(PDF)

S5 Table **Forward selection of variables in the redundancy analyses. Variables are ordered according to decreasing significance.** SeTem  =  sediment temperature, DisFo  =  distance to the forest, Win  =  winter, Sum  =  summer, DisCh  =  distance to the channels, WaLe  =  Water level, Dep  =  depth, Aut  =  autumn.
(PDF)
