# Supplementary material for: Edge Effects Are Important in Supporting Beetle Biodiversity in a Gravel-Bed River Floodplain
Source: PLoS One. 2014 Dec 29;9(12):e114415. doi: 10.1371/journal.pone.0114415 (PMC4278758; doi:10.1371/journal.pone.0114415)
Supplement: S1 Table — Full taxa list and pooled samples of beetles caught on the sediment surface and within subsurface sediments across a 200 m-wide gravel bar at the Tagliamento River (Italy). * species were only sampled on the sediment surface and can not or only partly fly (M. Kahlen, personal communication). (PDF) [file pone.0114415.s001.pdf]

Table S1. Full taxa list and pooled samples of beetles caught on the sediment surface and within subsurface sediments across a 200 m-wide gravel bar at the Tagliamento River (Italy). * species were only sampled on the sediment surface and can not or only partly fly (M. Kahlen, personal communication).

Taxa	Surface	Sediment	Only at channels	Only at the forest
	(No. of individuals)			
ANTHICIDAE				
Anthicus bimaculatus Illiger, 1801	3			
Anthicus luteicornis Schmidt, 1842	6			
Endomia occipitalis (Dufour, 1843)	2			
Endomia unifasciata (Bonelli, 1812)	1	2		
Mecynotarsus serricornis (Panzer, 1796)	5			
Omonadus floralis (Linnaeus, 1758)		1	1	
BYRRHIDAE				
Chaetophora spinosa (Rossi, 1794)	1		1	
CANTHARIDAE				
Cantharis montana Stierlin, 1889	1			
CARABIDAE				
Abax ater ssp. cf. lombardus Fiori, 1896*	2			2
Abax carinatus porcatus (Duftschmid, 1812)	10	1		11
Agonum afrum (Duftschmid, 1812)	3			3
Agonum fuliginosum (Panzer, 1809)	1			
Agonum muelleri (Herbst, 1784)	5			
Agonum sexpunctatum (Linnaeus, 1758)	1		1	

Taxa	Surface	Sediment	Only at channels	Only at the forest
	(No. of individuals)			
Agonum viduum (Panzer, 1796)	8			
Amara communis (Panzer, 1797)	2			2
Amara similata (Gyllenhal, 1810)	4			
Anchomenus dorsalis (Pontoppidan, 1763)	73			
Anisodactylus binotatus (Fabricius, 1787)	19			
Anisodactylus nemorivagus (Duftschmid, 1812)	6			
Asaphidion caraboides (Schrank, 1781)	373	1		
Asaphidion flavipes (Linnaeus, 1761)	75	1		
Asaphidion pallipes (Duftschmid, 1812)		1	1	
Bembidion azurescens (Dalla Torre, 1877)	3			
Bembidion bugnioni Daniel, 1902	10	12		
Bembidion cruciatum bualei Jacquelin du Val, 1852	48	6		
Bembidion decorum (Panzer, 1799)	9	2		
Bembidion distinguendum Jacquelin du Val, 1852	18	2		
Bembidion tarsicum Peyron, 1858	37	5		
Bembidion fasciolatum (Duftschmid, 1812)	113	53		
Bembidion foraminosum Sturm, 1825	3	7		
Bembidion fulvipes Sturm, 1827	264	23		
Bembidion lampros (Herbst, 1784)	1			1
Bembidion properans (Stephens, 1828)	1			1
Bembidion pseudosacendens Manderbach & Müller-Motzfeld, 2004	206	87		

Taxa	Surface	Sediment	Only at channels	Only at the forest
	(No. of individuals)			
Bembidion punctulatum Drapiez, 1821	6			
Bembidion pygmaeum (Fabricius, 1792)	7			7
Bembidion quadrimaculatum (Linnaeus, 1761)	4			
Bembidion ruficorne Sturm, 1825	7	4		
Bembidion scapulare oblongum Dejean, 1831	40	6		
Bembidion testaceum (Duftschmid, 1812)	21			
Bembidion tetracolum tetracolum Say, 1825	37	6		
Bembidion tibiale (Duftschmid, 1812)		1	1	
Bembidion varicolor Farbricius, 1803	1		1	
Broscus cephalotes Linnaeus, 1758*	19			
Calathus erratus (Sahlberg, 1827)	15			
Carabus cancellatus emarginatus Duftschmid, 1812*	3			
Carabus granulatus interstitialis Duftschmid, 1812*	12			
*Carabus violaceus ssp.**	1			1
Chlaenius nitidulus (Schrank, 1781)	35			
Chlaenius vestitus (Paykull, 1790)	4			
Cicindela hybrida transversalis Dejean in Latreille & Dejean, 1822	81			
Clivina collaris (Herbst, 1784)	3	11		
Cylindera arenaria (Fuesslin, 1775)	5			
Cylindera germanica (Linnaeus, 1758)*	9			
Diachromus germanus (Linnaeus, 1758)	1			1

Taxa	Surface	Sediment	Only at channels	Only at the forest
	(No. of individuals)			
Drypta dentata (Rossi, 1790)	1			1
Dyschirius abditus Fedorenko, 1993	1			
Dyschirius gracilis (Heer, 1837)	2			
Dyschirius intermedius Putzeys, 1846	1			
Dyschirius minutus Putzeys, 1867	1		1	
Dyschirius substriatus (Duftschmid, 1812)	3		3	
Elaphrus aureus Müller, 1821	220	2		
Harpalus luteicornis (Duftschmid, 1812)	1			1
Limodromus assimilis (Pykull, 1790)	24			24
Lionychus quadrillum (Duftschmid, 1812)	167	33		
Nebria brevicollis (Fabricius, 1792)	2			
Nebria picicornis (Fabricius, 1792)	145			
Notiophilus palustris (Duftschmid, 1812)	1			1
Omophron limbatum (Fabricius, 1776)	291	1		
Oodes helopioides (Fabricius, 1792)	9			
Paranchus albipes (Fabricius, 1796)	10			10
Paratachys bistriatus (Duftschmid, 1812)	1			
Paratachys micros (Fischer von Waldheim, 1828)	63	399		
Perileptus areolatus (Creutzer, 1799)	2	2		
Poecilus lepidus (Leske, 1785)	25	2		
Porotachys bisulcatus (Nicolai, 1822)	1	2		
Pseudoophonus griseus (Panzer, 1797)	5			

Taxa	Surface	Sediment	Only at channels	Only at the forest
	(No. of individuals)			
Pseudoophonus rufipes (De Geer, 1774)	1			1
Pterostichus melanarius (Illiger, 1798)	24	3		27
Pterostichus melas italicus (Dejean, 1828)*	1			1
Pterostichus niger (Schaller, 1783)	34	3		
Pterostichus nigrita (Paykull, 1790)	8			8
Pterostichus strenuus (Panzer, 1797)	1			1
Stenolophus teutonus (Schrank, 1781)	6			
Stomis pumicatus (Panzer, 1796)	1	3		4
Tachyura diabrachys Kolenati, 1845	2			
Tachyura parvula (Dejean, 1831)	1	1	1	
Tachyura sexstriata (Duftschimd, 1812)	21	17		
Thalassophilus longicornis (Sturm, 1825)	2	22		
CHRYSOMELIDAE				
Altica tamaricis Schrank, 1785	1			
Chaetocnema conducta (Motschulsky, 1838)	1			1
Chaetocnema hortensis (Geoffroy, 1785)	1		1	
Chaetocnema semicoerulea (Koch, 1803)	6	4		
Crepidodera aurata (Marsham, 1802)	1			1
Longitarsus nigrofasciatus (Goeze, 1777)	2			
Pachnephorus tessellatus (Duftschmidt, 1825)	1	1		
Phaedon armoraciae (Linnaeus, 1758)	6		6	
Phaedon cochleariae (Fabricius, 1792)	5			

Taxa	Surface	Sediment	Only at channels	Only at the forest
	(No. of individuals)			
Phaedon laevigatus (Duftschmid, 1825)	44			
Psylliodes napi (Fabricius, 1792)	1			1
Psylliodes picinus (Marsham, 1802)	5			5
COCCINNELLIDAE				
Hippodamia variegata (Goeze, 1777)	1			
CRYPTOPHAGIDAE				
Atomaria gottwaldi Johnson, 1971		1		
Atomaria gravidula Erichson, 1846	22			22
Atomaria impressa Erichson, 1846	1			
Atomaria lewisi Reitter, 1877	6			
Atomaria nigrirostris Stephens, 1830	686	296		
Atomaria plicata Reitter, 1875	1			
Atomaria pusilla (Paykull, 1798)		1	1	
LAEMOPHLOEIDAE				
Cryptolestes ferrugineus (Stephens, 1831)	3			
CURCULIONIDAE				
Acalyptus carpini (Fabricius, 1792)	1			1
Bagous glabrirostris (Herbst, 1795)	10			
Dorytomus taeniatus (Fabricius, 1781)	3			3
Hypothenemus eruditus Westwood, 1836	2			
Lepyrus palustris (Scopoli, 1763)	8			
Neophytobius quadrinodosus (Gyllenhal, 1813)	2		2	

Taxa	Surface	Sediment	Only at channels	Only at the forest
	(No. of individuals)			
Pseudomylloceus sinuatus Schoenherr, 1826	1			1
Tanymecus palliatus (Fabricius, 1787)*	2			
Trachyphloeus asperatus Boheman, 1843		1	1	
DRYOPIDAE				
Dryops ernesti Gozis, 1886	2			2
Dryops nitidulus (Heer, 1841)	6			
Dryops striatopunctatus (Heer, 1841)	3	30		
Dryops subincanus (Kuwert, 1890)	10	1		
Dryops viennensis (Laporte de Castelnau, 1840)	2	3		
DYTISCIDAE				
Agabus congener (Thunberg, 1794)	1			
Agabus didymus (Olivier, 1795)	1			1
Platambus maculatus (Linnaeus, 1758)	2			
Nebrioporus elegans (Panzer, 1794)	1			
Potamonectes depressus elegans Panzer, 1795		1	1	
ELATERIDAE				
Adrastus binaghii Leseigneur, 1969	2	2		
Adrastus lacertosus Erichson, 1841	2	1		
Adrastus pallens (Fabricius, 1792)		1		1
Agriotes litigiosus (Rossi, 1792)	1	1		
Betarmon bisbimaculatus (Fabricius, 1803)	2			2
Drasterius bimaculatus (Rossi, 1790)	8			

Taxa	Surface	Sediment	Only at channels	Only at the forest
	(No. of individuals)			
Negastrius sabulicola (Boheman, 1852)	1		1	
Paracardiophorus musculus (Erichson, 1840)	2			
Synaptus filiformis (Fabricius, 1781)	4	2		
Zorochros alysidotus (Kiesenwetter, 1858)	46	3		
Zorochros meridionalis (Laporte la Castelnau, 1840)	240	7		
Zorochros minimus (Boisduval & Lacordaire, 1835)		1		
Zorochros stibicki Leseigneur, 1970	18	7		
ELMIDAE				
Esolus parallelepipedus (Müller, 1806)		1	1	
GYRINIDAE				
Orectochilus villosus (Müller, 1776)	1		1	
HALIPLIDAE				
Brychius glabratus (Villa & Villa, 1835)	1			1
Haliplus laminatus (Schaller, 1783)	1			
Haliplus lineatocollis (Marsham, 1802)	3	5		
HISTERIDAE				
Hypocaccus rugifrons (Paykull, 1798)	15			
HYDRAENIDAE				
Hydraena sp.		1	1	
Ochthebius nobilis Villa & Villa, 1835		3	3	
GEORISSIDAE				
Georissus caelatus Erichson, 1847	3			

Taxa	Surface	Sediment	Only at channels	Only at the forest
	(No. of individuals)			
Georissus laesicollis Germar, 1831	7	6		
HYDROPHILIDAE				
Laccobius alternus Motschulsky, 1855	3			
Laccobius striatulus striatulus (Fabricius, 1801)	3		3	
LATRIDIIDAE				
Corticaria pubescens (Gyllenhal, 1827)	1		1	
Melanophthalma transversalis (Mannerheim, 1844)	3			
LEIODIDAE				
Agathidium atrum (Paykull, 1798)	1			1
Colon affine Sturm, 1839	1			1
Colon fuscicorne Kraatz, 1852	1			1
Leiodes carpathicus (Ganglbauer, 1896)	21			
Leiodes pallens (Sturm, 1807)	4			
Leiodes rotundatus (Erichson, 1845)	15			15
LIMNICHIDAE				
Limnichus incanus Kiesenwetter, 1851	10			
Limnichus sericeus (Duftschmid, 1825)	23			
LUCANIDAE				
Dorcus parallelipipedus (Linnaeus, 1758)	1			1
MONOTOMIDAE				
Monotoma longicollis (Gyllenhal, 1827)		86		
NITIDULIDAE				

Taxa	Surface	Sediment	Only at channels	Only at the forest
	(No. of individuals)			
Epuraea luteola Erichson, 1843	3			3
Glischrochilus quadrisignatus (Say, 1835)	9	7		
Stelidota geminata (Say, 1825)	60			
OEDERMERIDAE				
Anogcodes ferrugineus (Schrank, 1776)	1			1
PTILIIDAE				
Ptenidium longicorne Fuss, 1868	13	5		
Ptinella britannica Matthews, 1858		4		
Smicrus filicornis (Fairmaire & Laboulbène, 1855)	2		2	
MONOTOMIDAE				
Monotoma brevicollis Aubé, 1838	1			
Monotoma longicollis (Gyllenhal, 1827)	211			
Rhizophagus picipes (Olivier, 1790)	3	7		
SCARABAEIDAE				
Hoplia brunnipes Bonelli, 1812	2			2
Psammodius asper (Fabricius, 1775)	3	9		
Psammodius pierottii Pittino, 1979		1		
Rhyssemus limbolarius Petrovitz, 1963	52	2		
Serica brunnea (Linnaeus, 1758)	1			1
CURCULIONIDAE				
Xyleborus dispar (Fabricius, 1792)	8	1		
Xyleborus germanus (Blandford, 1894)	5	1		

Taxa	Surface	Sediment	Only at channels	Only at the forest
	(No. of individuals)			
Xyleborus saxesenii (Ratzeburg, 1837)	21	5		
SCYDMAENIDAE				
Chelonoidum latum (Motschulsky, 1851)	1	15		
SILPHIDAE				
Phosphuga atrata (Linnaeus, 1758)	6			6
STAPHYLINIDAE				
Aleochara haematoptera Kraatz, 1858	3	4		
Aloconota appulsa (Scriba, 1867)		1		
Aloconota cambrica (Wollaston, 1855)	3			
Aloconota eichhoffi (Scriba, 1867)	1			
Aloconota insecta (Thomson, 1856)	1	6		
Aloconota pfefferi (Roubal, 1929)	1	1		
Aloconota sulcifrons (Stephens, 1832)	2			
Amischa analis (Gravenhorst, 1802)	1			
Amauronyx maerkelii (Aubè, 1844)	4			4
Anotylus nitidulus (Gravenhorst, 1802)	1		1	
Anotylus rugosus (Fabricius, 1775)	104	305		
Anotylus tetracarinatus (Block, 1799)	2	2		4
Apimela macella (Erichson, 1839)		22		
Atheta autumnalis (Erichson, 1839)		4		
Atheta fungi (Gravenhorst, 1806)	1			1
Atheta pertyi (Heer, 1839)	1			1

Taxa	Surface	Sediment	Only at channels	Only at the forest
	(No. of individuals)			
Atheta sodalis (Erichson, 1837)	2			
Atheta triangulum (Kraatz, 1856)		5		
Bibloplectus sp.		2		
Bledius erraticus Erichson, 1839	1			1
Bledius littoralis Heer, 1839	1			
Brachygluta haematica (Reichenbach, 1816)	8	12		20
Brachygluta sp.	1			
Brachygluta trigonoprocta (Ganglbauer, 1895)	5			6
Brachygluta xanthoptera Reichenbach, 1816	61	44		
Bryaxis ullrichii (Motschulsky, 1851)	1			1
Bythinus reichenbachi (Machulka, 1928)	1	11		
Carpelimus bilineatus Stephens, 1834	61	9		
Carpelimus gracilis (Mannerheim, 1830)	39	106		
Carpelimus opacus (Baudi, 1848)	2			
Cypha pirazzolii Baudi, 1869	36	17		
Deleaster dichrous (Gravenhorst, 1802)	6	1		
Drusilla canaliculata (Fabricius, 1787)	2			2
Gabrius nigritulus (Gravenhorst, 1802)	1		1	
Gabrius osseticus (Kolenati, 1846)	3			3
Gabrius splendidulus (Gravenhorst, 1802)	2			2
Gabrius tirolensis (Luze, 1903)	1	1		
Geodromicus suturalis (Lacordaire, 1835)		1		

Taxa	Surface	Sediment	Only at channels	Only at the forest
	(No. of individuals)			
Hydrosmecta fluviatilis (Kraatz, 1854)	1		1	
Hydrosmecta gracilicornis (Erichson, 1839)	1	1		
Hydrosmecta haunoldiana (Bernhauer, 1914)		6		
Hydrosmecta quadraticeps Scheerpeltz, 1944	2			
Hydrosmecta valdieriana (Scheerpeltz, 1943)	1	2		
Ilyobates mech (Baudi, 1848)	1			1
Ischnosoma longicorne (Mäklin, 1847)	3			3
Ischnosoma splendidum (Gravenhorst, 1806)	2			2
Lathrobium brunnipes (Fabricius, 1793)	1			1
Lathrobium castaneipenne Kolenati, 1846	2			2
Lathrobium dilutum Erichson, 1839	1			2
Lathrobium ripicola Czwalina, 1888	1	12		
Leptacinus batychrus (Gyllenhal, 1827)		5		
Leptacinus sulcifrons (Stephens, 1833)	1			
Liogluta longiuscula (Gravenhorst, 1802)		2		2
Medon brunneus (Erichson, 1839)		1		1
Medon ripicola (Kraatz, 1854)	2		2	
Neobisnius lathrobioides (Baudi, 1848)	1	2		
Neobisnius procerulus (Gravenhorst, 1806)		1	1	
Neobisnius prolixus (Erichson, 1840)	1	7		
Neobisnius villosulus (Stephens, 1833)	26	10		
Ochthephilus angustatus (Erichson, 1840)	5	46		

Taxa	Surface	Sediment	Only at channels	Only at the forest
	(No. of individuals)			
Ochthephilus angustior (Bernhauer, 1943)	5	7		
Ochthephilus omalinus (Erichson, 1840)		43		
Ochthephilus praepositus Mulsant & Rey, 1878	1	8		
Ochthephilus rosenhaueri (Kiesenwetter, 1850)	2			
Ocypus nitens (Schrank, 1781)*	2			
Omalium caesum Gravenhorst, 1806	3			
Omalium rivulare (Paykull, 1789)		1		1
Paederidus rubrothoracicus (Goeze, 1777)	235	48		
Paederidus ruficollis (Fabricius, 1777)	34	11		
Paederus caligatus Erichson, 1840		1		1
Paederus limnophilus Erichson, 1840	13			
Paederus riparius (Linnaeus, 1758)	1	3		3
Parocyusa cingulata Kraatz, 1856		1		
Parocyusa longitarsis (Erichson, 1839)	1	3		
Parocyusa rubicunda (Erichson, 1837)	78	4		
Philonthus rotundicollis (Ménétriés, 1832)	19	2		21
Philonthus rubripennis (Stephens, 1832)	8			
Platystethus nitens (Sahlberg, 1832)	1			
Platydomene bicolor (Erichson, 1840)		76		
Platydomene picipes (Erichson, 1840)	1	16		
Platydomene springeri (Koch, 1937)	1	7		
Proteinus brachypterus (Fabricius, 1792)	2			

Taxa	Surface	Sediment	Only at channels	Only at the forest
	(No. of individuals)			
Proteinus ovalis Stephens, 1834	12	3		
Pseudomedon obsoletus (Nordmann, 1837)	1	1		
Quedius fuliginosus (Gravenhorst, 1802)	1			1
Rabigus tenuis (Fabricius, 1793)	28			
Scopaeus debilis Hochhuth, 1851	1	4		
Scopaeus gracilis (Sperk, 1835)	2			
Scopaeus laevigatus (Gyllenhal, 1827)		2		
Scopaeus sericans Mulsant & Rey, 1855		3	3	
Sepedophilus constans (Fowler, 1888)	2	1		
Sepedophilus marshami (Stephens, 1832)	5			5
Sepedophilus obtusus (Luze, 1902)	1			1
Sepedophilus pedicularius (Gravenhorst, 1802)	1		1	
Sepedophilus testaceus (Fabricius, 1793)	3			
Stenus boops ludmilae Hromádka, 1979	5			
Stenus carbonarius Gyllenhal, 1827	2			
Stenus fossulatus Erichson, 1840	1		1	
Stenus guttula Müller, 1821	3			
Stenus latens Puthz, 2003	7			
Stenus longipes Heer, 1839	17			
Stenus palposus Zetterstedt, 1838	1		1	
Stenus phyllobates miscellus Benick, 1925	1	1		
Stenus planifrons misael Bondroit, 1912	7			

Taxa	Surface	Sediment	Only at channels	Only at the forest
	(No. of individuals)			
Stenus ruralis Erichson, 1840	2			
Tachyporus abdominalis (Fabricius, 1781)	16			
Tachyporus atriceps Stephens, 1832	1			1
Tachyporus austriacus Luze, 1901	1			
Tachyusa balteata Erichson, 1839	30			
Taxicera deplanata (Gravenhorst, 1802)		1		
Taxicera dolomitana Bernhauer, 1900	77			
Thecturota marchii (Dodero, 1922)	1			
Thinobius crinifer Smetana, 1959	3	5		
Thinobius petzi Bernhauer, 1908	2	26		
Thinobius sp.	1			
Thinodromus dilatatus (Erichson, 1839)	9	5		
Tychobythinus sp.		10		
Xantholinus laevigatus Jacobsen, 1849	1			1
Xantholinus linearis (Olivier, 1795)	1			1
Zyras limbatus (Paykull, 1789)	13			
THROSCIDAE				
Trixagus dermestoides (Linnaeus, 1766)	2			
**Total individuals**	**5473**	**2187**	**272**	**127**
