# Supplementary material for: Edge Effects Are Important in Supporting Beetle Biodiversity in a Gravel-Bed River Floodplain
Source: PLoS One. 2014 Dec 29;9(12):e114415. doi: 10.1371/journal.pone.0114415 (PMC4278758; doi:10.1371/journal.pone.0114415)
Supplement: S2 Table — Species (No. of individuals) captured on the sediment surface (Surf.), within the subsurface sediments (Sed.), only at channel margins and at the riparian forest edge, respectively, that are of conservation concern (Status) according to Binot et al. [1] . Re = regionally extinct, CR = critically endangered, EN = endangered, VU = vulnerable, NT = near threatened, R = rare. (PDF) [file pone.0114415.s002.pdf]

Table S2. Species (No. of individuals) captured on the sediment surface (Surf.), within the subsurface sediments (Sed.), only at channel margins and at the riparian forest edge, respectively, that are of conservation concern (Status) according to Binot et al. [1]. Re = regionally extinct, CR = critically endangered, EN = endangered, VU = vulnerable, NT = near threatened, R = rare.

Taxa	Status	Surf.	Sed.	Only at channels	Only at the forest
		(No. of individuals)			
ANTHICIDAE					
Anthicus bimaculatus Illiger, 1801	VU	3			
Anthicus luteicornis Schmidt, 1842	CR	6			
Mecynotarsus serricornis (Panzer, 1796)	CR	5			
CARABIDAE					
Abax carinatus porcatus (Duftschmid, 1812)	VU	10	1		11
Anisodactylus nemorivagus (Duftschmid, 1812)	EN	6			
Asaphidion caraboides (Schrank, 1781)	CR	373	1		
Asaphidion pallipes (Duftschmid, 1812)	NT		1		
Bembidion azurescens (Dalla Torre, 1877)	EN	3			
Bembidion decorum (Panzer, 1799)	NT	9	2		
Bembidion distinguendum Jacquelin du Val, 1852	CR	18	2		
Bembidion fasciolatum (Duftschmid, 1812)	VU	113	53		
Bembidion foraminosum Sturm, 1825	Re	3	7		
Bembidion fulvipes Sturm, 1827	CR	265	23		
Bembidion punctulatum Drapiez, 1821	NT	6			
Bembidion pygmaeum (Fabricius, 1792)	NT	7			7

Taxa	Status	Surf.	Sed.	Only at channels	Only at the forest
		(No. of individuals)			
Bembidion ruficorne Sturm, 1825	NT	7	4		
Bembidion testaceum (Duftschmid, 1812)	NT	21			
Bembidion varicolor Farbricius, 1803	VU	1		1	
Broscus cephalotes Linnaeus, 1758	NT	19			
Carabus cancellatus emarginatus Duftschmid, 1812	NT	3			
Chlaenius nitidulus (Schrank, 1781)	VU	36			
Clivina collaris (Herbst, 1784)	NT	3	11		
Cylindera germanica (Linnaeus, 1758)	CR	9			
Dyschirius abditus Fedorenko, 1993	R	1			
Dyschirius intermedius Putzeys, 1846	VU	1			
Dyschirius substriatus (Duftschmid, 1812)	R	3		3	
Elaphrus aureus Müller, 1821	EN	220	2		
Lionychus quadrillum (Duftschmid, 1812)	NT	167	33		
Omophron limbatum (Fabricius, 1776)	NT	291	1		
Perileptus areolatus (Creutzer, 1799)	VU	2	2		
Poecilus lepidus (Leske, 1785)	NT	25	2		
Thalassophilus longicornis (Sturm, 1825)	EN	2	22		
CHRYSOMELIDAE					
Altica tamaricis Schrank, 1785	VU	1			
Chaetocnema semicoerulea (Koch, 1803)	VU	6	4		
Pachnephorus tessellatus Duftschmidt, 1825	EN	1	1		
CRYPTOPHAGIDAE					

Taxa	Status	Surf.	Sed.	Only at channels	Only at the forest
		(No. of individuals)			
Atomaria gottwaldi Johnson, 1971	CR		1		
Atomaria gravidula Erichson, 1846	VU	22			22
Atomaria impressa Erichson, 1846	VU	1			
Atomaria plicata Reitter, 1875	EN	1			
DRYOPIDAE					
Dryops nitidulus (Heer, 1841)	CR	6			
Dryops striatopunctatus (Heer, 1841)	CR	11	30		
Dryops subincanus (Kuwert, 1890)	VU	5	1		
Dryops viennensis (Laporte de Castelnau, 1840)	EN	2	3		
ELATERIDAE					
Betarmon bisbimaculatus (Fabricius, 1803)	EN	2			2
Drasterius bimaculatus (Rossi, 1790)	Re	8			
Negastrius sabulicola (Boheman, 1851)	VU	1		1	
Paracardiophorus musculus (Erichson, 1840)	EN	2			
HYDRAENIDAE					
Ochthebius nobilis Villa & Villa, 1835	CR		3		
GEORISSIDAE					
Georissus laesicollis Germar, 1831	CR	7	6		
HYDROPHILIDAE					
Laccobius alternus Motschulsky, 1855	EN	3			
LEIODIDAE					
Colon fuscicorne Kraatz, 1852	Re	1			1

Taxa	Status	Surf.	Sed.	Only at channels	Only at the forest
		(No. of individuals)			
Leiodes carpathicus Ganglbauer, 1896	EN	21			
Leiodes pallens (Sturm, 1807)	EN	4			
Leiodes rotundatus (Erichson, 1845)	EN	15			15
PTILIIDAE					
Ptinella britannica Matthews, 1858	VU		4		
Smicrus filicornis (Fairmaire & Laboulbene, 1855)	VU	2		2	
SCYDMAENIDAE					
Chelonoidum latum (Motschulsky, 1851)	Re	1	15		
STAPHYLINIDAE					
Aleochara haematoptera Kraatz, 1858	VU	3	4		
Aloconota appulsa (Scriba, 1867)	EN		1		
Aloconota eichhoffi (Scriba, 1867)	EN	1			
Aloconota pfefferi (Roubal, 1929)	VU	1	1		
Aloconota planifrons (Waterhouse, 1864)	VU	3			
Amauronyx maerkelii (Aubè, 1844)	EN	4			4
Apimela macella (Erichson, 1839)	VU		22		
Bledius littoralis Heer, 1839	VU	1			
Brachygluta trigonoprocta (Ganglbauer, 1895)	Re	6			6
Brachygluta xanthoptera Reichenbach, 1816	VU	62	44		
Bythinus reichenbachi (Machulka, 1928)	CR	2	11		
Cypha pirazzolii Baudi, 1869	CR	40	17		
Gabrius tirolensis (Luze, 1903)	VU	1	1		

Taxa	Status	Surf.	Sed.	Only at channels	Only at the forest
		(No. of individuals)			
Lathrobium castaneipenne Kolenati, 1846	EN	2			2
Lathrobium dilutum Erichson, 1839	VU	2			2
Lathrobium ripicola Czwalina, 1888	VU	1	12		
Medon ripicola (Kraatz, 1854)	VU	2		2	
Neobisnius prolixus (Erichson, 1840)	VU	1	7		
Paederus caligatus Erichson, 1840	VU		1		
Parocyusa cingulata Kraatz, 1856	VU		1		
Scopaeus sericans Mulsant & Rey, 1855	VU	1	3	4	
Sepedophilus constans (Fowler, 1888)	EN	2	1		
Stenus longipes Heer, 1839	VU	15			
Stenus palposus Zetterstedt, 1838	EN	1		1	
Stenus phyllobates miscellus Benick, 1925	EN	1	1		
Stenus planifrons misael Bondroit, 1912	EN	7			
Tachyusa balteata Erichson, 1839	VU	30			
Taxicera dolomitana (Bernhauer, 1900)	CR	77			
Thinobius crinifer Smetana, 1959	EN	3	5		
Thinodromus dilatatus (Erichson, 1839)	VU	9	5		
**Total individuals**		**2039**	**372**	**14**	**7**

References

1. Binot M, Bless R, Boye P, Gruttke H, Pretscher P (1998) Rote Liste gefährdeter Tiere Deutschlands. Bonn-Bad Godesberg: Bundesamt für Naturschutz.
