# Supplementary material for: Edge Effects Are Important in Supporting Beetle Biodiversity in a Gravel-Bed River Floodplain
Source: PLoS One. 2014 Dec 29;9(12):e114415. doi: 10.1371/journal.pone.0114415 (PMC4278758; doi:10.1371/journal.pone.0114415)
Supplement: S3 Table — Eigenvalues of the partial RDA, and the cumulative percentage of variance explained by significant axes A) for all species and B) for species of conservation concern. (PDF) [file pone.0114415.s003.pdf]

Table S3. Eigenvalues of the partial RDA, and the cumulative percentage of variance explained by significant axes A) for all species and B) for species of conservation concern.

A)

	All species								
Axes	1	2	3	4	5	6	7	8	9
Eigenvalues	0.046	0.031	0.020	0.011	0.009	0.006	0.005	0.002	0.001
Cumulative % variance	4.6	7.7	9.7	10.8	11.7	12.3	12.8	13.0	13.1
F ratio	27.8**	18.6**	11.8**	6.5**	5.5**	3.4**	3.1**	1.1	0.7

Significance: ** ≤ 0.001

B)

	Species of conservation concern (red list species)								
Axes	1	2	3	4	5	6	7	8	9
Eigenvalues	0.066	0.033	0.016	0.007	0.006	0.004	0.002	0.002	0.001
Cumulative % variance	6.6	9.9	11.4	12.1	12.7	13.0	13.2	13.4	13.5
F ratio	26.6**	13.4**	6.3**	2.9**	2.3*	1.4	0.8	0.8	0.4

Significance: ** ≤ 0.001, * ≤ 0.01
