# Supplementary material for: Edge Effects Are Important in Supporting Beetle Biodiversity in a Gravel-Bed River Floodplain
Source: PLoS One. 2014 Dec 29;9(12):e114415. doi: 10.1371/journal.pone.0114415 (PMC4278758; doi:10.1371/journal.pone.0114415)
Supplement: S4 Table — The contribution of spatial, environmental, and temporal variables to the variation in the two species matrices (all species and species of conservation concern only) explained by the partial RDA. R2 adjusted with Ezekiel's formula [1]. (PDF) [file pone.0114415.s004.pdf]

Table S4. The contribution of spatial, environmental, and temporal variables to the variation in the two species matrices (all species and species of conservation concern only) explained by the partial RDA. R^2 adjusted with Ezekiel´s formula [1].

Variables		All species			Species of conservation concern		
	df	F	R^2_{adj}	Variation (%)	F	R^2_{adj}	Variation (%)
Spatial	3	10.1*	0.051	5.1	5.8*	0.047	4.5
Environmental	3	5.0*	0.055	2.4	2.7*	0.023	2.1
Temporal	3	4.8*	0.056	2.5	1.5*	0.013	1.2

Significance: * ≤ 0.001

References

1. Ezekiel M (1930) Methods of correlation analysis. New York: Jon Wiley and Sons.
