# Supplementary material for: Edge Effects Are Important in Supporting Beetle Biodiversity in a Gravel-Bed River Floodplain
Source: PLoS One. 2014 Dec 29;9(12):e114415. doi: 10.1371/journal.pone.0114415 (PMC4278758; doi:10.1371/journal.pone.0114415)
Supplement: S5 Table — Forward selection of variables in the redundancy analyses. Variables are ordered according to decreasing significance. SeTem = sediment temperature, DisFo = distance to the forest, Win = winter, Sum = summer, DisCh = distance to the channels, WaLe = Water level, Dep = depth, Aut = autumn. (PDF) [file pone.0114415.s005.pdf]

Table S5. Forward selection of variables in the redundancy analyses. Variables are ordered according to decreasing significance. SeTem = sediment temperature, DisFo = distance to the forest, Win = winter, Sum = summer, DisCh = distance to the channels, WaLe = Water level, Dep = depth, Aut = autumn.

Total species			Red list species		
Variables	F value	P value	Variables	F value	P value
SeTem	10.3	0.001*	SeTem	4.5	0.001*
DisFo	9.5	0.001*	Win	3.5	0.001*
Win	7.0	0.001*	DisFo	3.3	0.001*
Rain	6.3	0.001*	Sum	2.3	0.002
Sum	5.4	0.001*	WaLe	2.2	0.005
DisCh	4.9	0.001*	Dep	2.1	0.007
Spring	4.1	0.001*	Spr	2.0	0.009
WaLe	3.1	0.001*	DisCh	2.0	0.009
Dep	3.0	0.001*	Rain	1.8	0.015
Aut	2.0	0.005	Aut	1.7	0.017

Significance: * ≤ 0.001
